# Integrative multi-omics and experimental analyses implicate PTK2 as a lorazepam-associated biomarker and potential therapeutic target in ovarian cancer

**DOI:** 10.3389/fphar.2025.1744802

**Published:** 2026-01-23

**Authors:** Xia Hong, Xingjun He, Suli He

**Affiliations:** 1 Department of Obstetrics and Gynecology, Affiliated Hospital of Yangzhou University, Yangzhou, China; 2 Department of Urology, Affiliated Hospital of Yangzhou University, Yangzhou, China

**Keywords:** drug repurposing, immunemicroenvironment, lorazepam, multi-omics, ovarian cancer, Ptk2, spatial transcriptomics

## Abstract

**Background:**

Ovarian cancer remains one of the most lethal gynecologic malignancies, characterized by high recurrence rates and chemoresistance. Recent advances suggest that neuroactive drugs such as lorazepam may exert off-target molecular effects beyond the central nervous system. However, their pharmacological relevance in tumor biology remains largely unexplored. This study aimed to identify and validate shared molecular targets of lorazepam and ovarian cancer, uncovering novel biomarkers and therapeutic mechanisms.

**Methods:**

Potential lorazepam targets were retrieved from the CTD, STITCH, and SwissTargetPrediction databases and cross-referenced with ovarian cancer–related genes from GeneCards. Bioinformatic analyses were conducted, including target screening, protein–protein interaction network construction, Lasso–Cox modeling, and functional enrichment. We further performed immune infiltration profiling, single-cell and spatial transcriptomics analyses, followed by experimental validation using qPCR, CCK-8, colony formation, wound-healing, and flow cytometry assays.

**Results:**

Fifty-one overlapping genes were identified between lorazepam and ovarian cancer, with PTK2 emerging as a central hub gene. PTK2 overexpression was significantly associated with poor prognosis, enhanced immune activation, stromal remodeling, and metabolic reprogramming. Spatial transcriptomics revealed PTK2 enrichment in malignant and fibroblast-rich regions. Functional assays confirmed that stable PTK2 knockdown inhibited proliferation, colony formation, and migration while promoting apoptosis in ovarian cancer cells.

**Conclusion:**

Our findings suggest that PTK2 may act as a putative oncogenic mediator and potential pharmacological link between lorazepam signaling and ovarian cancer progression. PTK2 functions as a biomarker and druggable target that integrates tumor growth, immune modulation, and metabolic adaptation. Targeting PTK2 may provide a promising therapeutic strategy for precision oncology and rational drug repurposing.

## 1 Introduction

Ovarian cancer (OV) is one of the most lethal malignancies among women worldwide, characterized by late-stage diagnosis, extensive metastasis, and poor therapeutic response (Arnaoutoglou et al., 2023; Torre et al., 2018; Gong et al., 2022). Despite advances in surgery and chemotherapy, the 5-year survival rate for advanced OV remains below 30%, largely due to tumor heterogeneity and therapy resistance (Bowtell et al., 2015; Zhang et al., 2025; Zhao et al., 2023a). Therefore, identifying novel molecular biomarkers and therapeutic targets that can improve early diagnosis and guide precision treatment remains a critical unmet need.

Lorazepam, a widely used benzodiazepine, is clinically prescribed to alleviate anxiety, insomnia, and chemotherapy-induced nausea in cancer patients (Hesketh et al., 2020). Beyond its neuropsychiatric effects, emerging evidence indicates that benzodiazepines can influence cancer progression by modulating cell proliferation, apoptosis, and immune responses through γ-aminobutyric acid (GABA)–related or off-target signaling pathways (Li et al., 2023; Huang et al., 2023; Tian and Kaufman, 2023). In ovarian cancer, components of the GABA_A pathway (e.g., GABRP) have been linked to aggressive behavior and epigenetic dysregulation, supporting a potential mechanistic connection worth exploring (Juvale et al., 2021; Sung et al., 2017). However, the potential molecular targets of lorazepam in ovarian cancer remain largely unexplored, and their pharmacological and mechanistic relevance to tumor biology is poorly understood (Zalewska et al., 2016; Iorio et al., 2016).

Protein tyrosine kinase 2 (PTK2), also known as focal adhesion kinase (FAK), is a cytoplasmic non-receptor tyrosine kinase that regulates cell adhesion, migration, and survival *via* integrin and growth factor signaling (Sato et al., 2022). Aberrant PTK2 activation has been implicated in various cancers, including breast, lung, and hepatocellular carcinoma, where it promotes tumor proliferation, epithelial–mesenchymal transition (EMT), angiogenesis, and resistance to targeted therapy. In ovarian cancer, PTK2 overexpression has been linked to metastasis and platinum resistance, but its mechanistic role in modulating the tumor microenvironment (TME), immune infiltration, and metabolic reprogramming remains incompletely defined (Ozmadenci et al., 2022; Zhang et al., 2024). Moreover, recent pharmacogenomic analyses suggest that PTK2 may serve as a downstream effector or interaction node for several psychotropic and kinase-targeting drugs, indicating a possible pharmacological intersection between lorazepam signaling and oncogenic pathways.

With the rapid advancement of high-throughput multi-omics and artificial intelligence (AI)-driven analytical tools, integrative pharmacogenomic studies have become powerful strategies to identify drug–target–disease relationships and reveal novel therapeutic mechanisms (Subramanian et al., 2017; Corsello et al., 2020; Song et al., 2023; Jiang C. et al., 2024). Combining multi-database target prediction, transcriptomic profiling, and functional validation allows for systematic discovery of drug-related oncogenic drivers (Yuan et al., 2023; Zhao et al., 2023b; Peng et al., 2022; Wei et al., 2022; Feng et al., 2025). Furthermore, single-cell and spatial transcriptomics technologies have enabled unprecedented resolution in delineating the spatial organization of tumor ecosystems, providing critical insights into how gene expression patterns influence cellular heterogeneity, stromal remodeling, and immune–tumor interactions within the TME (Sood et al., 2004; Jiang L. et al., 2024).

In this study, we aimed to elucidate the potential pharmacological link between lorazepam and ovarian cancer progression by identifying common molecular targets and characterizing their biological functions. Through multi-database integration and protein–protein interaction (PPI) network analysis, we identified PTK2 as a central hub gene shared between lorazepam targets and ovarian cancer–related genes. Multi-omics analyses—including bulk transcriptomics, gene set enrichment analysis (GSEA), immune infiltration profiling, and single-cell and spatial transcriptomics—were applied to comprehensively characterize the signaling, metabolic, and immunological landscape associated with PTK2. Finally, *in vitro* validation using stable PTK2 knockdown models confirmed its role in promoting ovarian cancer cell proliferation, migration, and survival.

Given the frequent clinical use of lorazepam in oncology patients, clarifying its molecular influence on tumor biology is both timely and clinically relevant. Our work addresses this gap by systematically connecting pharmacological data with tumor multi-omics and experimental evidence.

## 2 Methods

### 2.1 Target screening and cross-target identification

Known targets of lorazepam were retrieved from the Comparative Toxicogenomics Database (CTD) (https://ctdbase.org/) (Davis et al., 2021) and the STITCH database (http://stitch.embl.de/) (Szklarczyk et al., 2016). The CTD and STITCH databases provide experimentally supported or literature-curated target information, whereas SwissTargetPrediction predicts targets computationally based on ligand–target similarity. The SMILES structure of lorazepam was obtained from PubChem (https://pubchem.ncbi.nlm.nih.gov) (Kim et al., 2023) and submitted to the SwissTargetPrediction platform (http://www.swisstargetprediction.ch) for target prediction (Daina et al., 2019). All identified targets were merged and deduplicated to generate a comprehensive set of potential lorazepam-related targets. Subsequently, the GeneCards database (https://www.genecards.org/) was queried using the keyword “ovarian cancer,” with the organism restricted to *Homo sapiens*, to obtain ovarian cancer–associated genes. A Venn diagram was constructed using the SangerBox platform (http://www.sangerbox.com/) to determine the intersection between the lorazepam target set and the ovarian cancer gene set (Shen et al., 2022). The overlapping targets were defined as candidate genes for downstream analyses.

### 2.2 PPI network construction and core target identification

The intersecting genes (official gene symbols) were imported into the STRING (Search Tool for the Retrieval of Interacting Genes/Proteins) database (https://string-db.org/) to construct a protein–protein interaction (PPI) network, setting the species to *Homo sapiens* and the minimum required interaction score to 0.9 (high confidence). The resulting network data were visualized using Cytoscape software (version 3.8.2) (Szklarczyk et al. 2023). To identify key nodes, the cytoHubba plugin was employed, ranking nodes based on the Maximal Clique Centrality (MCC) algorithm (Chin et al., 2014). The top 10 genes with the highest MCC scores were defined as core (hub) targets.

### 2.3 Machine learning screening of prognostic genes

Based on the expression profiles of core target genes, Lasso-Cox regression analysis was conducted using the glmnet package in R (version 4.3.2) to identify optimal prognosis-related genes (Friedman et al., 2010). The family parameter was set to “cox,” with a maximum iteration limit of 1000. Ten-fold cross-validation was performed using the cv.glmnet function to determine the optimal penalty parameter (*λ*).

### 2.4 Survival and meta-analysis

Kaplan–Meier (K–M) survival analysis was carried out using the survival package in R. Patients were stratified into high- and low-risk groups based on the median risk score, and group differences in disease-free interval (DFI), disease-specific survival (DSS), overall survival (OS), and progression-free interval (PFI) were compared using the log-rank test. To comprehensively evaluate the prognostic utility of the risk model, meta-analysis was performed using the meta package in R (Balduzzi et al., 2019). The inverse variance method under a random-effects model was applied to pool log-hazard ratios (logHR) derived from univariate Cox analyses across the four survival endpoints (DFI, DSS, OS, PFI).

### 2.5 Functional enrichment analysis

Functional enrichment analysis of the core targets was conducted using the Bioinformatics platform (https://www.bioinformatics.com.cn/) (Yu et al., 2012). Gene Ontology (GO) enrichment was performed across three categories—Biological Process (BP), Cellular Component (CC), and Molecular Function (MF)—and the top 10 most significantly enriched terms (ranked by P-values) were visualized as bar plots (Chen et al., 2024). In parallel, Kyoto Encyclopedia of Genes and Genomes (KEGG) pathway enrichment was carried out *via* the SangerBox platform (http://www.sangerbox.com/) (Kanehisa and Sato, 2020), and the top 10 pathways with the highest significance were presented using Circos plots (Zhou et al., 2019). Enrichment analysis was performed using KEGG and GO datasets with the ranked log2FC values as input, and significance was defined at adjusted *p* < 0.05 (Benjamini–Hochberg FDR correction).

### 2.6 Expression and prognostic validation of key genes

The Gene Expression Profiling Interactive Analysis 2 (GEPIA2) web server (http://gepia2.cancer-pku.cn/) (Tang et al., 2019) was employed to compare expression differences of the core target genes between TCGA tumor samples and GTEx normal tissues (both expressed as TPM). The survival analysis module in GEPIA2 was further used to assess the effect of PTK2 expression (high vs. low) on overall survival (OS) and disease-free survival (DFS) in ovarian cancer patients. To further validate PTK2 expression at the protein level, UALCAN (CPTAC module) was used to compare PTK2 protein abundance between normal ovarian tissues and primary ovarian tumors, and results were presented as Z-values. In addition, representative PTK2 immunohistochemistry (IHC) images for ovarian cancer and normal ovary tissues were retrieved from the Human Protein Atlas (HPA) database for qualitative comparison of staining patterns.

### 2.7 Pathway and metabolic analysis of PTK2 in ovarian cancer

Ovarian cancer samples (TCGA-OV) were divided into high and low PTK2 expression groups, defined as the top and bottom 50% of PTK2 expression levels, respectively. Differential expression analysis between the two groups was performed using the limma package in R, and log2 fold-change (log2FC) or Z-score values were calculated for each gene (Ritchie et al., 2015). For genes represented by multiple probes, the median expression value was used to minimize outlier influence and ensure robust quantification. A ranked gene list was used for Gene Set Enrichment Analysis (GSEA) with the clusterProfiler package (Yu et al., 2012), based on the Hallmark and KEGG gene sets. Normalized Enrichment Scores (NES) were calculated, and multiple hypothesis testing was applied to identify pathways significantly associated with PTK2 expression. Additionally, single-sample enrichment analysis was performed using the GSVA package (Hänzelmann et al., 2013) with the “zscore” parameter on KEGG metabolic gene sets. Samples with the highest 50% PTK2 expression were defined as the high-expression group, and the lowest 50% as the low-expression group. The limma package was used to compare GSVA scores between groups to identify PTK2-related metabolic features.

### 2.8 Immune infiltration analysis

To systematically assess the relationship between PTK2 and the tumor immune microenvironment, Spearman correlation analysis was performed to evaluate associations between PTK2 expression and the scores of each step of the Cancer–Immunity Cycle, as well as inter-step autocorrelations. Visualization was conducted using the linkET package (Kalinka and Tomancak, 2011) in a combined network–matrix format. Data on immune-stimulatory and -inhibitory genes, chemokines, and human leukocyte antigen (HLA) genes were obtained from the TISIDB database (http://cis.hku.hk/TISIDB/) (Ru et al., 2019). Samples were divided into high and low PTK2 expression groups, and gene expression differences were analyzed using the Wilcoxon rank-sum test, with the results visualized as heat maps showing the average expression of immune-related molecules. To explore the association between PTK2 and immune response or genomic state, samples were divided into quartiles (Q1–Q4) based on PTK2 expression, following the immune response and genomic state scoring system described by Thorsson et al. (2018). The average value of each score (excluding missing values) was calculated across quartile groups and visualized using pheatmap. Furthermore, immune infiltration data for TCGA-OV were obtained from the TIMER2.0 database (Li et al., 2020). Spearman correlation analysis was conducted across multiple algorithms to evaluate the relationships between PTK2 expression and immune cell abundance, with results visualized using heat maps for cross-method comparison. Finally, samples were classified into high and low expression groups according to the median PTK2 expression level. The Wilcoxon rank-sum test was used to identify significantly altered immune cell populations, which were visualized in heat maps ordered by PTK2 expression, where color intensity represents the relative abundance of immune cells.

### 2.9 Single-cell expression profiling of PTK2

The publicly available ovarian cancer single-cell RNA sequencing dataset OV_EMTAB8107, which includes the largest number of untreated cells to date, was analyzed. Uniform Manifold Approximation and Projection (UMAP) was applied for dimensionality reduction and visualization of the transcriptomic landscape. Given the high dropout rates inherent to single-cell sequencing, the Nebulosa package (Alquicira-Hernandez and Powell, 2021) was utilized to perform weighted kernel density estimation, integrating local similarities among cells to recover missing gene expression signals and improve visualization accuracy. Data preprocessing, normalization, and clustering were performed using the Seurat R package (Hao et al., 2021). UMAP clustering successfully distinguished major cellular populations, including malignant epithelial cells, fibroblasts, endothelial cells, immune cells, monocytes/macrophages, and plasma cells, with further refinement into subclusters based on lineage-specific markers. To evaluate the differences in PTK2 expression across cell types, the Kruskal–Wallis rank-sum test was employed. This non-parametric method assesses median differences among multiple independent groups without assuming data normality. Expression levels of PTK2 were quantified in each cell type, and statistical significance among cell populations was visualized using violin plots, box plots, and bar charts.

### 2.10 Spatial transcriptomics analysis of PTK2 expression in ovarian cancer

To characterize the spatial distribution of PTK2 in ovarian cancer tissues, spatial transcriptomics analysis was performed on ovarian cancer tissue sections (GSM6177614) using the Sparkle database, which integrates multi-cancer 10x Visium datasets and provides standardized preprocessing pipelines. This adaptive threshold ensured adequate group size and statistical stability when the number of fully malignant spots was below 30. Raw expression counts were normalized using the NormalizeData function to remove technical variation across samples. PTK2 expression was visualized with Seurat’s SpatialFeaturePlot function, which integrates gene expression data with spatial coordinates to generate color-coded spot maps displaying tissue-level expression patterns. The SpatialPlot function was applied to map the distribution of dominant cell types across tissue sections. Spearman correlation analysis quantified relationships both between different cell type abundances and between cell type composition and PTK2 expression, with the linkET package used for integrated network–matrix visualization of correlation direction, strength, and significance. Spatial region segmentation was based on deconvolution results: spots with malignant cell proportion = 1 were defined as malignant regions, 0 as normal regions, and intermediate values as mixed regions. When fully malignant spots were fewer than 30, an adaptive strategy defined spots with malignant proportion >0.5 as highly malignant (hMal), 0 as non-malignant (nMal), and the remainder as low malignant (lMal); if group sizes were still insufficient, spots with proportion >0 were unified as malignant (Mal) and 0 as non-malignant (nMal). To further refine boundaries, Cottrazm was employed to cluster morphology-adjusted expression matrices and identify tumor cores based on copy-number variation (CNV) profiles; using a hexagonal lattice framework, adjacent spots were extended outward from the tumor centroid to delineate boundary (Bdy) and adjacent non-malignant (nMal) regions through the Cottrazm-boundarydefine function. Inter-regional PTK2 expression differences were statistically assessed with the Wilcoxon rank-sum test (wilcox.test in R), which accommodates non-normal data distributions. Finally, mean expression values across defined regions were visualized using the fromto package (dplot5 function) as bar plots to intuitively represent the spatial distribution pattern of PTK2 within ovarian cancer tissue.

### 2.11 Cell lines and culture conditions

Three human cell lines were used in this study: SK-OV-3 and A2780 ovarian cancer cell lines, and the HOSEpiC human ovarian surface epithelial cell line. SK-OV-3 and A2780 cells were cultured in RPMI-1640 medium supplemented with 10% fetal bovine serum (FBS) and 1% penicillin–streptomycin, while HOSEpiC cells were maintained in serum-free MCDB 105 medium supplemented with 5% FBS. All cell lines were incubated at 37 °C in a humidified atmosphere containing 5% CO_2_, and routinely tested to ensure the absence of *mycoplasma* contamination.

### 2.12 Quantitative real-time PCR (qPCR)

Total RNA was extracted using a commercial RNA extraction kit (Takara, Japan), and RNA concentration and purity were assessed using a NanoDrop 2000 spectrophotometer (Thermo Fisher Scientific, USA). Complementary DNA (cDNA) was synthesized using the PrimeScript RT Reagent Kit (Thermo Fisher Scientific, USA) according to the manufacturer’s instructions. Quantitative real-time PCR was performed using SYBR Green PCR Master Mix (Thermo Fisher Scientific, USA) on a CFX96 Touch Real-Time PCR Detection System (Bio-Rad, USA). The ΔΔCt method was used to determine relative mRNA expression, with GAPDH serving as the internal control for normalization. All experiments were conducted in triplicate, and relative expression levels were calculated as 2^−ΔΔCt^ values.

### 2.13 Cell viability assay (CCK-8)

Cell viability was determined using the Cell Counting Kit-8 (CCK-8, Dojindo, Japan) following the manufacturer’s protocol. SK-OV-3 and A2780 cells were seeded into 96-well plates at a density of 2 × 10^3^ cells per well and incubated for 24, 48, and 72 h. Subsequently, 10 μL of CCK-8 reagent was added to each well, and the plates were incubated for 2 h at 37 °C. Absorbance was measured at 450 nm using a microplate reader (BioTek, USA). Cell viability was expressed as a percentage of the control group, calculated as the ratio of the optical density (OD) of treated to control cells.

### 2.14 Colony formation assay

The clonogenic ability of ovarian cancer cells was assessed using the colony formation assay. A total of 1,000 cells were seeded per well in six-well plates and cultured for 10 days in complete RPMI-1640 medium containing 10% FBS, with medium refreshed every 3 days. At the end of incubation, colonies were fixed and stained with 0.5% crystal violet solution for 30 min at room temperature, then rinsed with PBS to remove excess dye. Colonies containing more than 50 cells were counted under a light microscope (Leica, Germany). Colony formation efficiency was calculated as the number of colonies divided by the number of seeded cells.

### 2.15 Wound healing assay

The wound healing assay was used to evaluate the migratory capacity of ovarian cancer cells. SK-OV-3 and A2780 cells were plated in six-well plates at a density of 1 × 10^6^ cells per well and grown to approximately 90% confluence. A straight scratch was created using a sterile 200 μL pipette tip, followed by washing with PBS to remove debris. Cells were incubated in serum-free medium for 24 and 48 h, and wound closure was observed and photographed using an inverted optical microscope (Olympus, Japan). Cell migration was quantified by measuring the wound width at each time point and calculating the percentage of wound closure relative to time 0.

### 2.16 Flow cytometric analysis of apoptosis

Cell apoptosis was analyzed using the Annexin V–FITC/PI apoptosis detection kit (Thermo Fisher Scientific, Cat. No. V13242) according to the manufacturer’s protocol. Briefly, cells were harvested by trypsinization, washed twice with PBS, and resuspended in binding buffer. Each sample was incubated with 5 μL Annexin V–FITC and 5 μL propidium iodide (PI) for 15 min at room temperature in the dark. After staining, 400 μL of binding buffer was added, and samples were analyzed on a BD FACSCanto II flow cytometer (BD Biosciences, USA). Apoptotic cells were classified as early apoptotic (Annexin V^+^/PI^−^) or late apoptotic/necrotic (Annexin V^+^/PI^+^) using FlowJo software (FlowJo LLC, USA). The apoptosis rate was calculated as the proportion of Annexin V–positive cells.

### 2.17 Statistical analysis

All statistical analyses were performed using R software (version 4.3.2) and GraphPad Prism 9.0 (GraphPad Software, USA). Data are presented as mean ± standard deviation (SD) from at least three independent experiments unless otherwise specified. Comparisons between two groups were conducted using the Student’s t-test for normally distributed data or the Wilcoxon rank-sum test for non-parametric data. For comparisons among multiple groups, one-way analysis of variance (ANOVA) followed by Tukey’s *post hoc* test was used for normally distributed data, while the Kruskal–Wallis test followed by Dunn’s multiple-comparison test was applied for non-parametric data. Spearman’s correlation analysis was employed to assess relationships between gene expression levels and immune or cellular features. Kaplan–Meier survival curves were generated to evaluate overall survival (OS), disease-free survival (DFS), disease-specific survival (DSS), and progression-free interval (PFI), and differences between groups were compared using the log-rank test. A two-tailed P-value <0.05 was considered statistically significant, with the following notation used in all figures: *p* < 0.05 (*), *p* < 0.01 (**), and *p* < 0.001 (***).

## 3 Results

### 3.1 Identification of shared targets between ovarian cancer and lorazepam

**FIGURE 1:**
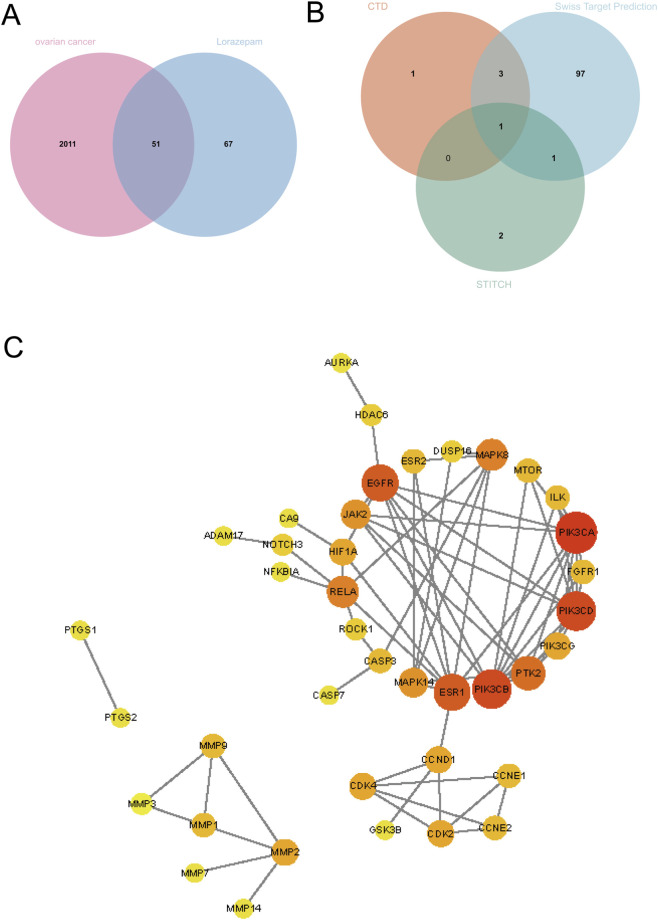
Identification of common targets of lorazepam and ovarian cancer, and construction of the PPI network. **(A)** Venn diagram showing the overlap between ovarian cancer–related genes retrieved from the GeneCards database and potential lorazepam targets. **(B)** Venn diagram summarizing the sources of lorazepam targets, integrating predictions from the CTD, SwissTargetPrediction, and STITCH databases. **(C)** Protein–protein interaction (PPI) network of the 51 overlapping genes, constructed using the STRING database (minimum required interaction score = 0.9) and visualized in Cytoscape. Node size and color intensity correspond to the Maximal Clique Centrality (MCC) scores from cytoHubba analysis, with larger and darker nodes representing higher network centrality.

From the GeneCards database, we retrieved 2,011 genes associated with ovarian cancer. Meanwhile, integration of the CTD, STITCH, and SwissTargetPrediction databases identified 67 putative targets of lorazepam (Figure 1B). Cross-analysis *via* SangerBox revealed 51 overlapping genes between lorazepam-related targets and ovarian cancer-associated genes (Figure 1A), which were subsequently used for downstream analysis.

### 3.2 PPI network construction and hub gene identification

**FIGURE 2:**
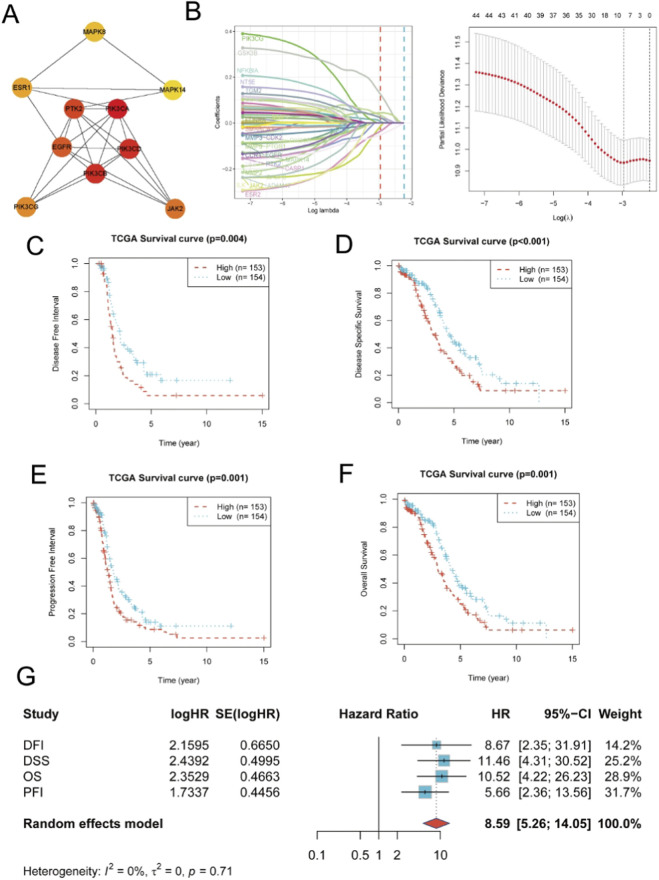
Hub gene identification, prognostic model construction, and validation. **(A)** PPI subnetwork of the top 10 hub genes identified by the MCC algorithm. **(B)** Lasso–Cox regression analysis for prognostic gene selection. The left panel shows the Lasso coefficient profiles of the 10 hub genes as a function of log(λ), while the right panel displays the partial likelihood deviance from 10-fold cross-validation, with vertical dashed lines indicating the optimal λ value. **(C–F)** Kaplan–Meier survival curves comparing high-risk (red dashed) and low-risk (blue solid) groups in the TCGA cohort, stratified by the median risk score: **(C)** disease-free interval (DFI), **(D)** disease-specific survival (DSS), **(E)** progression-free interval (PFI), and **(F)** overall survival (OS). **(G)** Forest plot of meta-analysis results integrating Cox regression outcomes across the four survival endpoints (DFI, DSS, OS, PFI) as an internal consistency check rather than a cross-study meta-analysis. The pooled hazard ratio (HR) and 95% confidence interval (CI) were calculated under a random-effects model; the diamond denotes the combined HR estimate.

The 51 shared targets were imported into the STRING platform to build a high-confidence PPI network (Figure 1C), which was visualized in Cytoscape. Using the MCC algorithm implemented in the cytoHubba plugin, the top 10 hub genes were identified: PTK2, ESR1, PIK3R1, PIK3CA, PIK3CB, PIK3CD, MAPK1, MAPK3, MAPK8, and EGFR (Figure 2A).

### 3.3 Functional enrichment analysis

**FIGURE 3:**
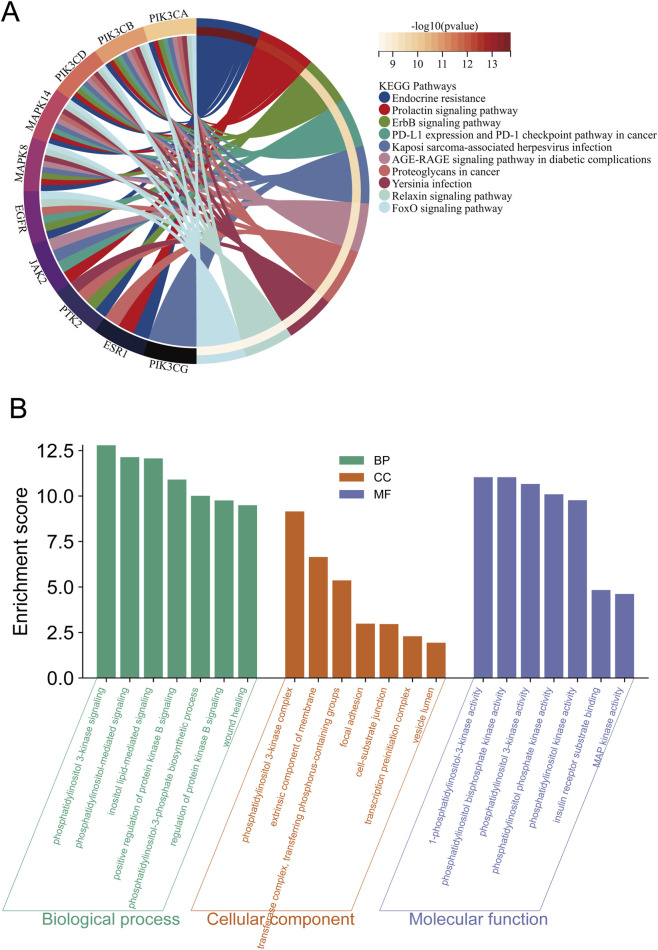
Functional enrichment analysis of hub genes. **(A)** Chord diagram illustrating the top 10 enriched KEGG pathways for the 10 hub genes. Gene symbols are shown on the left, while the corresponding pathways are listed on the right. The outer color gradient (red scale) indicates statistical significance (−log_10_ *p*-value), with darker hues denoting greater enrichment. **(B)** Bar plot of Gene Ontology (GO) enrichment analysis, presenting the top 10 significantly enriched terms in biological process (BP, green), cellular component (CC, orange), and molecular function (MF, purple) categories.

GO and KEGG enrichment analyses were performed to characterize the biological functions of the hub genes. KEGG analysis (Figure 3A) showed significant enrichment in cancer-relevant pathways, including PI3K-Akt signaling, endocrine resistance, and proteoglycans in cancer. GO enrichment (Figure 3B) revealed that, in terms of Biological Processes (BP), hub genes were primarily involved in phosphatidylinositol-mediated signaling; in the Cellular Component (CC) category, they were enriched in phosphatidylinositol 3-kinase complex; and in the Molecular Function (MF) category, they were mainly associated with phosphatidylinositol kinase activity.

### 3.4 Development of a prognostic risk model based on hub genes

To determine prognostic relevance, we constructed a Lasso-Cox regression model in the TCGA cohort. Ten-fold cross-validation identified the optimal *λ* value, yielding eight genes with non-zero coefficients: EGFR, ESR1, MAPK1, MAPK8, PIK3CA, PIK3CB, PIK3R1, and PTK2 (Figure 2B). Risk scores were then calculated based on the expression levels and corresponding coefficients of these genes.

### 3.5 Model validation and meta-analysis

Patients were divided into high- and low-risk groups by median risk score. Kaplan–Meier curves (Figures 2C–F) showed that patients in the high-risk group experienced significantly worse DFI (p = 0.004), DSS (*p* < 0.001), PFI (*p* < 0.001), and OS (*p* < 0.001). To further assess robustness, a meta-analysis of the four Cox models was performed, yielding a pooled HR of 8.59 (95% CI: 5.26–14.05) with no significant heterogeneity (*I* ^2^ = 0%, *p* = 0.71) (Figure 2G), indicating strong predictive performance.

### 3.6 Expression and prognostic significance of PTK2

**FIGURE 4:**
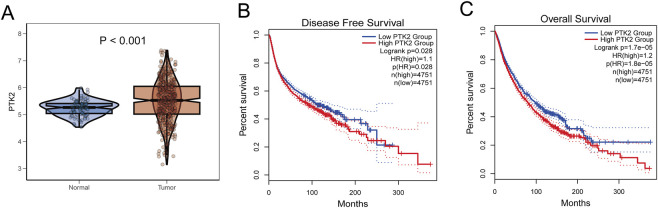
Differential expression and prognostic significance of PTK2. **(A)** Violin plot comparing PTK2 transcript levels (TPM) between normal tissues (GTEx, blue) and ovarian cancer tissues (TCGA, red); *p* < 0.001 indicates a significant difference. **(B,C)** Kaplan–Meier survival analysis of PTK2 expression using GEPIA2. Patients were divided into high- and low-expression groups (red vs. blue) based on the median PTK2 level. **(B)** Disease-free survival (DFS) and **(C)** overall survival (OS) curves are displayed, with the corresponding hazard ratios (HR) and log-rank p-values indicated.

Among the eight model genes, PTK2 was further evaluated in detail using GEPIA2 (TCGA-OV and GTEx cohorts), which confirmed that PTK2 expression was significantly higher in ovarian cancer tissues compared with normal tissues (*p* < 0.001) (Figure 4A). GEPIA2 survival analysis revealed that high PTK2 expression was significantly associated with worse DFS (log-rank p = 0.028, HR = 1.1) and OS (log-rank *p* = 1.7 × 10^−5^, HR = 1.2) (Figures 4B,C), suggesting its potential role as an unfavorable prognostic biomarker.

### 3.7 PTK2-mediated signaling pathway activation and metabolic reprogramming

**FIGURE 5:**
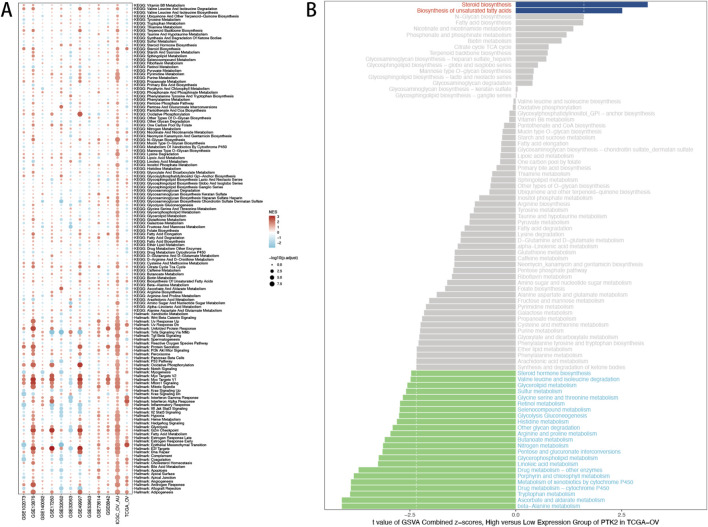
Pathway Analysis of PTK2 in Ovarian Cancer (OV). **(A)** Gene Set Enrichment Analysis (GSEA) results for PTK2. **(B)** GSVA analysis of metabolic pathways between high and low PTK2 expression groups.

To explore the biological programs associated with PTK2, we compared pathway and metabolic signatures between PTK2-high and PTK2-low tumors. High PTK2 expression is associated with prominent pathway enrichment features, particularly those related to cell adhesion, extracellular matrix remodeling, and epithelial–mesenchymal transition, including Epithelial Mesenchymal Transition, Angiogenesis, Focal Adhesion, and TGF-β Signaling. Moreover, activation of IL6/JAK/STAT3 and TNFA Signaling *via* NF-κB suggests that PTK2 may promote tumor–stroma interaction and immune microenvironment modulation (Figure 5A). In contrast, metabolic pathways associated with fatty acid metabolism, oxidative phosphorylation, and glutathione metabolism are enriched in the PTK2 low-expression group, indicating a more flexible metabolic state and better oxidative stress coping capacity. GSVA analysis further revealed that amino acid metabolic pathways (e.g., Lysine degradation, Arginine and proline metabolism, and Glycine/serine/threonine metabolism) and lipid metabolic pathways (Glycerophospholipid metabolism, Fatty acid degradation) are upregulated in the PTK2 high-expression group, suggesting enhanced membrane biosynthesis and amino acid–driven metabolic reprogramming. Conversely, detoxification- and oxidative stress–related pathways (Drug metabolism–cytochrome P450, Metabolism of xenobiotics by cytochrome P450, and Retinol metabolism) are significantly downregulated, implying reduced metabolic detoxification capacity and increased tolerance to microenvironmental stress (Figure 5B). Collectively, these results suggest that high PTK2 expression drives ovarian cancer progression through coordinated signaling activation, metabolic reprogramming, and stromal reinforcement, providing mechanistic support for PTK2 as a potential therapeutic target.

### 3.8 PTK2 promotes immune activation and stromal remodeling within the tumor microenvironment

**FIGURE 6:**
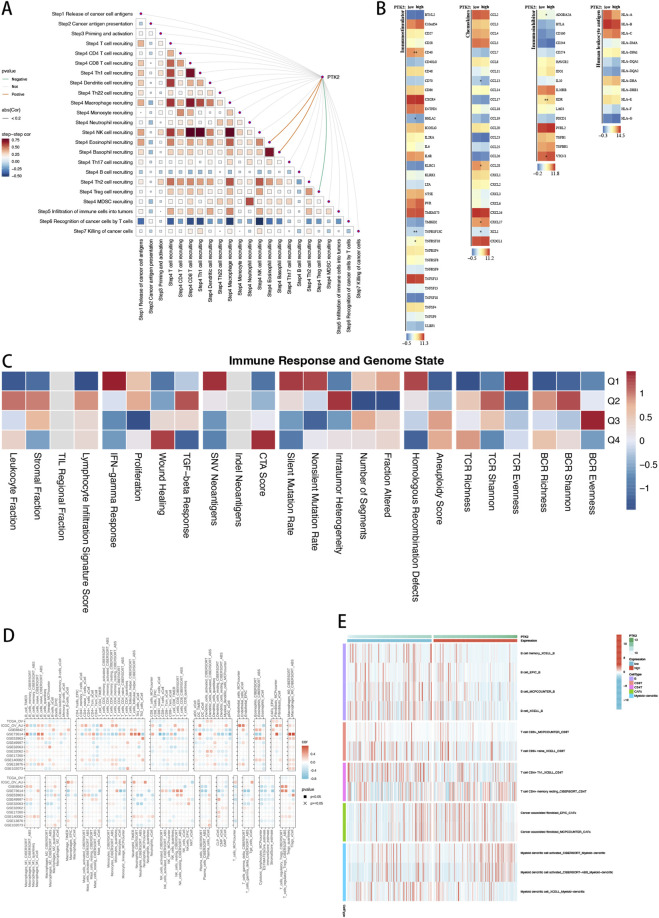
Immune Infiltration Analysis of PTK2 in Ovarian Cancer (OV). **(A)** Spearman correlation analysis between PTK2 expression levels and each step of the cancer–immunity cycle. **(B)** Differences in immune response and genomic status scores among PTK2 expression quartile groups, with color intensity representing row-standardized relative levels. **(C)** Spearman correlations between PTK2 expression and immune cell abundance evaluated using multiple algorithms; colors indicate correlation direction and strength, and “×” denotes non-significance. **(D)** Heat map showing significant differences in immune cell infiltration levels between high and low PTK2 expression groups; color represents cell abundance, with red indicating higher levels. **(E)** Heatmap of significantly different immune cell infiltration levels in high/low PTK2 expression groups, where colors indicate cell abundance levels, with red denoting higher content.

In ovarian cancer, elevated PTK2 expression is closely linked to remodeling of the tumor immune microenvironment. PTK2 expression shows strong positive correlations with multiple key steps of the cancer–immunity cycle, including immune cell recruitment, intratumoral infiltration, tumor antigen recognition, and effector cell–mediated cytotoxicity. In contrast, correlations with immunosuppressive processes—such as regulatory T-cell (Treg) and myeloid-derived suppressor cell (MDSC) recruitment—are weak, suggesting that PTK2 is preferentially associated with an immune-activated rather than an immunosuppressive phenotype (Figure 6A). Consistently, immune-stimulatory molecules (e.g., CD27, CD40, and TNFRSF family members) and pro-inflammatory chemokines (CCL5, CXCL16, CX3CL1) are significantly upregulated in the PTK2 high-expression group, while immune checkpoint genes (PDCD1, LAG3, HAVCR2) are also elevated, indicating feedback activation of inhibitory pathways. Meanwhile, enhanced expression of HLA-I/II molecules suggests improved antigen presentation and increased tumor immune visibility (Figure 6B). At the interface between immune response and genomic state, PTK2 high expression is associated with elevated wound-healing signatures, enhanced TGF-β signaling, stromal enrichment, and proliferative activity, accompanied by greater chromosomal instability and reduced TCR/BCR diversity. These features suggest that PTK2 may promote a coupled process of stromal remodeling and clonal evolution, which could limit sustained lymphocyte infiltration (Figure 6C). Multi-algorithm immune infiltration analyses further confirmed strong and consistent positive correlations between PTK2 and effector immune populations—including CD8^+^ T cells, Th1/memory CD4^+^ T cells, activated NK cells, M1 macrophages, and dendritic cells—whereas associations with M2 macrophages were weak. The convergence of results across different analytical methods underscores the robustness of these findings (Figure 6D). Moreover, the PTK2 high-expression group exhibited marked enrichment of memory B cells, CD8^+^ T cells, Th1/memory CD4^+^ T cells, and activated dendritic cells, along with a pronounced increase in cancer-associated fibroblasts (CAFs). As different deconvolution algorithms (e.g., TIMER, EPIC, quanTIseq) may yield slightly variable estimates, we focused on consistent directional trends across methods to ensure robustness. These patterns suggest that PTK2 orchestrates a tumor microenvironment characterized by strong immune activation coupled with intensive stromal remodeling (Figure 6E).

### 3.9 Single-cell spatial distribution and cell type–specific expression of PTK2

**FIGURE 7:**
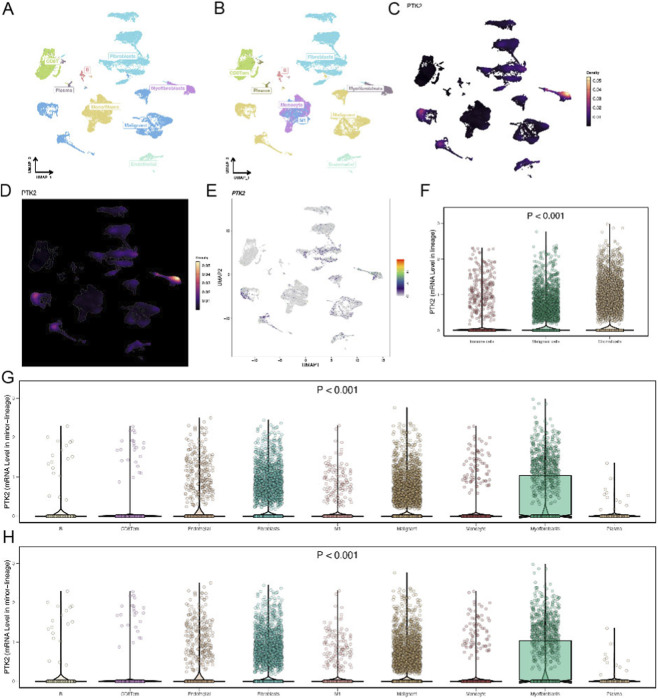
Single-cell spatial distribution and cell type–specific expression of PTK2 in ovarian cancer. **(A,B)** Each dot represents a single cell. The UMAP distribution reflects transcriptional similarity among cells, with colors indicating either cell type or gene expression level. **(C–E)** The color scale on the right represents gene expression intensity, where red denotes high expression and gray indicates low expression. **(F–H)** PTK2 gene expression across different cell types.

To determine which cell populations predominantly express PTK2, we assessed its distribution across major cell lineages at single-cell resolution. In the UMAP embedding, ovarian cancer cells displayed a clear spatial segregation, with distinct boundaries among malignant, stromal, and immune compartments (Figures 7A,B). At the single-cell level, PTK2 exhibited a pronounced spatially restricted expression pattern, being primarily enriched in malignant epithelial cells and stromal fibroblast populations (especially fibroblasts and myofibroblasts), while showing low expression in immune cells such as T cells, monocytes, and plasma cells (Figures 7C–E). This expression landscape suggests that PTK2 may participate in processes such as tumor cell adhesion, migration, and extracellular matrix remodeling, and its upregulation in tumor-associated fibroblasts further supports a pivotal role in microenvironmental reorganization. The Kruskal–Wallis rank-sum test revealed significant differences in PTK2 expression among cell lineages (*p* < 0.001). Overall, PTK2 expression was markedly higher in stromal cells—particularly fibroblasts and myofibroblasts—as well as in malignant epithelial cells—compared to immune cells, with myofibroblasts showing the highest expression levels, indicating a potential role in matrix remodeling and cell adhesion. Conversely, PTK2 expression was relatively low and heterogeneous in T cells, B cells, monocytes/macrophages, and plasma cells. Fine-grained minor-lineage analysis revealed modest PTK2 upregulation in M1 macrophages and a subset of CD8^+^ central memory T cells (CD8^+^ Tcm), suggesting limited engagement of PTK2 under immune-activated conditions. Collectively, these findings indicate that PTK2 is predominantly localized within malignant and stromal compartments of the ovarian cancer microenvironment and may contribute to tumor progression through the regulation of cell adhesion and stromal architecture (Figures 7F–H).

### 3.10 Spatial transcriptomic analyses delineate the cellular architecture and expression dynamics of PTK2 within ovarian cancer tissue

**FIGURE 8:**
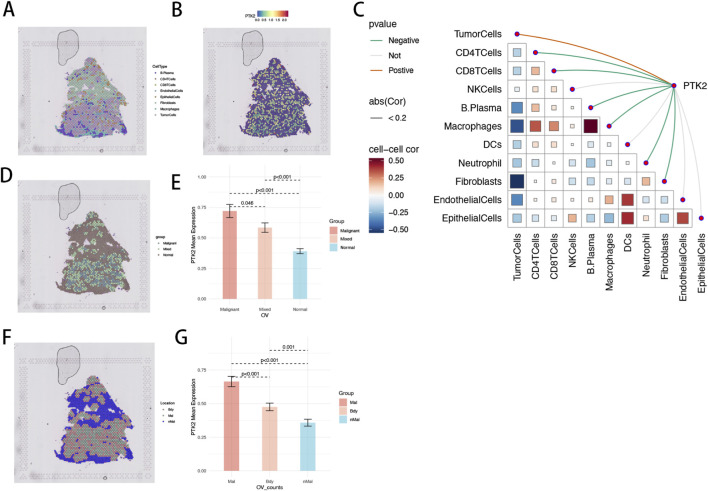
Spatial Transcriptomic Characterization of PTK2 in Ovarian Cancer. **(A)** The spatial cell-type map illustrates the organization of major cellular populations, including malignant, stromal, and immune compartments. **(B)** Spatial feature plots reveal the heterogeneous distribution of PTK2 expression, predominantly localized to tumor-enriched regions. **(C)** The spatial correlation network depicts relationships between PTK2 expression and different cell type abundances, highlighting its strong association with malignant and stromal cells. **(D,E)** Quantitative spatial expression analyses demonstrate significantly elevated PTK2 levels in malignant regions relative to mixed, boundary, and normal areas, underscoring its tumor-specific spatial pattern and potential role in microenvironmental remodeling. **(F,G)** Bar graphs comparing mean PTK2 expression across tumor core (Mal), boundary (Bdy), and normal (nMal) regions, with statistical significance indicated.

To further characterize the spatial organization of PTK2 within the tumor ecosystem, we analyzed its expression using spatial transcriptomics. Spatial transcriptomic analysis revealed a well-organized cellular architecture in ovarian cancer tissue. Malignant cells were predominantly concentrated in the tumor core, fibroblasts and endothelial cells localized at the periphery, and immune cells—including T cells, macrophages, and plasma cells—distributed mainly in stromal and marginal regions. This configuration reflects the characteristic “tumor-core enrichment, stromal encapsulation, and immune embedding” organization of ovarian cancer, providing spatial insight into its microenvironmental composition (Figure 8A). PTK2 displayed pronounced spatial heterogeneity, with its high-expression zones overlapping tumor cell–dense regions, while expression declined markedly in normal and stromal areas. This spatial pattern suggests that PTK2 is mainly expressed by tumor cells and may be functionally involved in adhesion, migration, and extracellular matrix remodeling. These findings align with single-cell results showing high PTK2 expression in malignant and fibroblast populations, reinforcing its importance in tumor progression and microenvironmental remodeling (Figure 8B). Correlation analysis further revealed a strong positive relationship between PTK2 expression and tumor cell abundance, a moderate correlation with fibroblasts, and weak or nonsignificant correlations with immune cells such as T cells, NK cells, and macrophages (Figure 8C). This indicates that PTK2 predominantly originates from structural cell populations, contributing to adhesion and matrix remodeling processes. The consistency between correlation and spatial expression patterns reinforces PTK2’s central functional role in maintaining tissue architecture and tumor dynamics. Regional classification based on malignant cell proportion showed that PTK2 expression was significantly higher in malignant regions compared with mixed and normal regions (*p* < 0.001) (Figures 8D,E). Furthermore, spatial segmentation using the Cottrazm algorithm demonstrated that PTK2 expression was highest in the tumor core (Mal), moderate at the tumor boundary (Bdy), and lowest in normal regions (nMal) (p < 0.001) (Figures 8F,G). This gradient pattern reveals a distinct spatial hierarchy of PTK2 expression: high expression in the tumor core likely supports structural integrity and adhesion, while moderate upregulation at the boundary suggests a transitional role in tumor–stroma interaction and local invasion. Collectively, PTK2 exhibited distinct spatial specificity in ovarian cancer, with elevated expression closely associated with the tumor core, structural cell populations, and matrix remodeling. These findings highlight PTK2’s pivotal role in spatially orchestrating tumor progression and provide new evidence for its potential as a therapeutic target in ovarian cancer.

### 3.11 PTK2 promotes ovarian cancer cell proliferation

**FIGURE 9:**
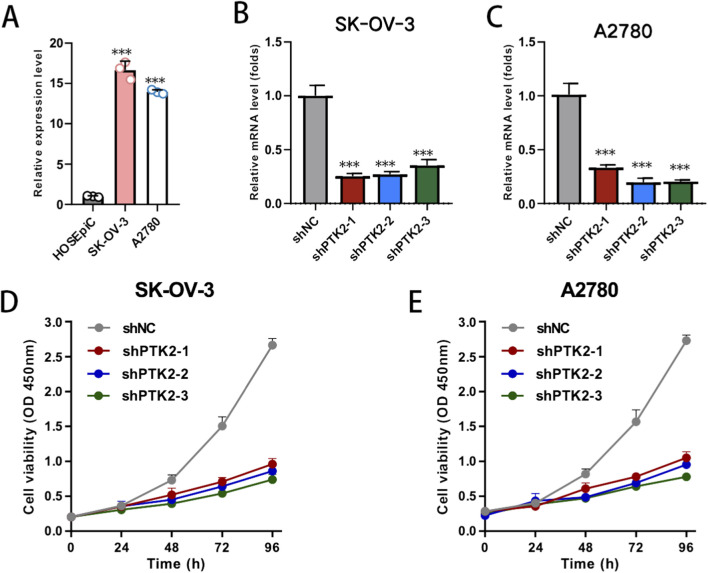
PTK2 knockdown suppresses ovarian cancer cell proliferation. **(A)** Quantitative PCR analysis showing PTK2 mRNA expression in normal ovarian epithelial cells (HOSEpiC) and ovarian cancer cell lines (SK-OV-3 and A2780). **(B,C)** Validation of stable PTK2 knockdown (shPTK2) efficiency in SK-OV-3 and A2780 cells. **(D,E)** CCK-8 assay showing that PTK2 knockdown significantly reduced the proliferation rate of SK-OV-3 and A2780 cells at 24, 48, and 72 h compared with control cells. Data are presented as mean ± SD; *p* < 0.05, *p* < 0.01, *p* < 0.001.

To further support PTK2 upregulation beyond transcriptomic analyses, we assessed PTK2 expression at the protein level using public proteomic and IHC resources. UALCAN analysis based on CPTAC samples showed that PTK2 protein abundance was higher in primary ovarian tumors than in normal tissues (Supplementary Figure 1A). Consistently, immunohistochemistry images from the Human Protein Atlas (HPA) demonstrated stronger PTK2 staining in ovarian cancer tissue compared with normal ovary tissue (Supplementary Figures 1B,C), providing orthogonal evidence of PTK2 upregulation at the protein level in tumors. To further validate the biological role of PTK2 in ovarian cancer, we next examined its expression across normal and malignant ovarian cell lines. Quantitative PCR analysis revealed that PTK2 mRNA expression was markedly upregulated in ovarian cancer cell lines (SK-OV-3 and A2780) compared with normal ovarian surface epithelial cells (HOSEpiC) (Figure 9A, *p* < 0.001). To investigate its functional role, stable PTK2 knockdown (shPTK2) cell lines were generated in SK-OV-3 and A2780 cells, and knockdown efficiency was confirmed by qPCR (Figures 9B,C, *p* < 0.001). Functional assays demonstrated that PTK2 knockdown significantly suppressed cell viability and proliferation, as evidenced by the CCK-8 assay, where shPTK2 cells exhibited a time-dependent decrease in optical density compared with controls (Figures 9D,E, *p* < 0.01). These findings suggest that PTK2 functions as a pro-proliferative regulator in ovarian cancer cells, promoting tumor cell growth and malignant progression.

### 3.12 PTK2 knockdown suppresses clonogenic growth, induces apoptosis, and impairs migration in ovarian cancer cells

**FIGURE 10:**
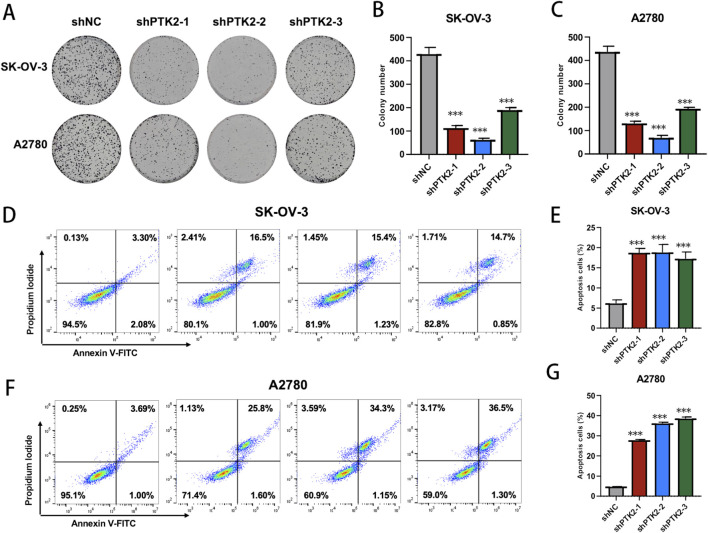
PTK2 knockdown impairs clonogenic growth and induces apoptosis in ovarian cancer cells. **(A–C)** Colony formation assay in SK-OV-3 and A2780 cells showing that shPTK2 significantly decreases colony number and size compared with control. **(D,E)** Annexin V–FITC/PI flow cytometry in SK-OV-3 cells demonstrating increased apoptotic fraction after PTK2 knockdown. **(F,G)** Annexin V–FITC/PI flow cytometry in A2780 cells showing a similar increase in apoptosis upon PTK2 depletion.

**FIGURE 11:**
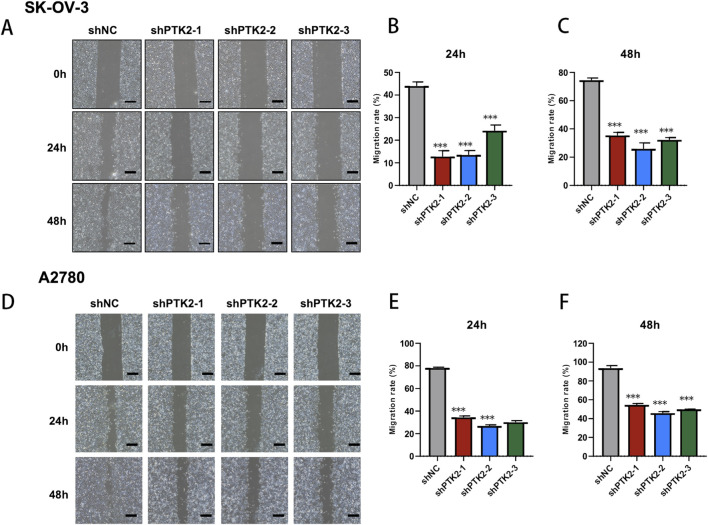
PTK2 knockdown suppresses ovarian cancer cell migration. **(A–C)** Wound-healing assay in SK-OV-3 cells showing that shPTK2 significantly delays wound closure at 24 and 48 h compared with control cells. **(D–F)** Wound-healing assay in A2780 cells showing consistent inhibition of cell migration after PTK2 knockdown.

To further validate the functional role of PTK2 in ovarian cancer, we performed *in vitro* assays following stable PTK2 knockdown (shPTK2) in SK-OV-3 and A2780 cells. Colony formation assays revealed that PTK2 depletion markedly reduced clonogenic capacity, with significantly fewer and smaller colonies formed compared to the control group (Figures 10A–C, *p* < 0.01). In parallel, Annexin V–FITC/PI flow cytometric analysis showed a pronounced increase in apoptotic cell populations in both SK-OV-3 and A2780 cells after PTK2 knockdown (Figures 10D–G, p < 0.01), indicating that PTK2 contributes to cell survival and anti-apoptotic regulation. Moreover, wound-healing assays demonstrated that PTK2 silencing substantially inhibited cell migration, as evidenced by slower wound closure at 24 and 48 h in both SK-OV-3 and A2780 cells compared with controls (Figures 11A–F, *p* < 0.01). Together, these results indicate that PTK2 enhances the proliferative, survival, and migratory abilities of ovarian cancer cells, whereas its knockdown suppresses these malignant phenotypes.

## 4 Discussion

In this study, we systematically identified PTK2 (protein tyrosine kinase 2) as a key molecular target potentially linking lorazepam pharmacology with ovarian cancer progression. Through an integrated multi-omics and experimental approach, PTK2 was found to be markedly overexpressed in ovarian cancer tissues and to play a pivotal role in promoting tumor cell proliferation, migration, and survival. Multi-dimensional analyses, including transcriptomic, single-cell, and spatial transcriptomics, revealed that PTK2 expression is predominantly localized in malignant epithelial and fibroblast populations, where it contributes to remodeling of the tumor immune microenvironment (TME) and metabolic adaptation. Collectively, these findings suggest that PTK2 functions not only as a tumor-promoting kinase but also as a potential pharmacological mediator involved in lorazepam-related signaling.

PTK2, also known as focal adhesion kinase (FAK), acts as a central regulator of integrin-mediated signal transduction and cytoskeletal organization (Sulzmaier et al., 2014; Schaller, 2010; Lee et al., 2015). Consistent with previous reports implicating FAK in metastasis and therapeutic resistance (Lee et al., 2015), our study demonstrated that PTK2 expression was significantly elevated in ovarian cancer tissues and cell lines compared with normal epithelial cells. Functional assays confirmed that PTK2 silencing inhibited proliferation and colony formation, reduced wound-healing capacity, and induced apoptosis in SK-OV-3 and A2780 cells (Tai et al., 2015). These findings align with the established role of PTK2 in activating downstream signaling cascades, including PI3K–AKT, MAPK, and STAT3, which promote epithelial–mesenchymal transition (EMT), angiogenesis, and survival under stress conditions (Mitra et al., 2005). Enrichment analyses further indicated that PTK2 upregulation was associated with enhanced activation of EMT, focal adhesion, and angiogenesis pathways, supporting its central function in tumor invasion and progression (Zhao and Guan, 2009).

Beyond its canonical role in adhesion and migration, PTK2 also appears to exert substantial influence on the immune landscape of ovarian cancer (Jiang et al., 2016; Serrels et al., 2015). Our immune infiltration analysis revealed strong correlations between PTK2 expression and multiple steps of the cancer-immunity cycle, including T-cell activation, chemokine signaling, and effector-cell recruitment. Tumors with high PTK2 expression displayed concurrent upregulation of immune-stimulatory molecules such as CD27, CD40, and TNFRSF members, as well as immune checkpoints including PDCD1, LAG3, and HAVCR2, suggesting a dynamic interplay between immune activation and regulatory suppression (Lee et al., 2015). Notably, infiltration of CD8^+^ T cells, NK cells, and M1 macrophages was positively associated with PTK2 expression, indicating an immune-active yet partially exhausted microenvironment. This observation is consistent with recent studies demonstrating that pharmacologic inhibition of FAK can modulate the tumor immune milieu and potentiate responses to immune checkpoint blockade. Thus, targeting PTK2 may represent a strategy to restore immune surveillance and enhance the efficacy of immunotherapy in ovarian cancer.

Metabolic reprogramming represents another critical dimension of PTK2-mediated oncogenic regulation (Zhao and Guan, 2009). Our gene set enrichment analysis showed that PTK2-high tumors exhibited significant activation of amino acid and lipid metabolism pathways, including lysine, arginine, and fatty acid metabolism, while detoxification and oxidative stress–related processes were downregulated. These findings suggest that PTK2 promotes metabolic flexibility, allowing tumor cells to maintain energy homeostasis and biosynthetic activity under nutrient-limited or hypoxic conditions (Sieg et al., 2000). Spatial transcriptomic analysis further revealed that PTK2 is enriched in tumor core and boundary regions with elevated stromal and wound-healing signatures, implicating its role in extracellular matrix (ECM) remodeling (Mitra and Schlaepfer, 2006). Such stromal remodeling not only provides a supportive niche for tumor expansion but also forms physical barriers that hinder immune cell infiltration, thereby contributing to immune evasion and therapeutic resistance (Serrels et al., 2015). Taken together, these data highlight the multifaceted role of PTK2 in coordinating metabolic and structural dynamics within the TME.

Although lorazepam is primarily recognized for its anxiolytic effects through γ-aminobutyric acid (GABA) receptor modulation, emerging evidence indicates that benzodiazepines may have broader biological effects extending beyond the central nervous system (Maaser et al., 2002; Bhattacharya et al., 2021). Our analysis revealed an intersection between lorazepam-associated targets and ovarian cancer–related genes, with PTK2 identified as a shared node. This suggests that lorazepam, or its downstream molecular interactions, may modulate oncogenic pathways involving PTK2 signaling. Several epidemiological studies have reported altered survival outcomes in cancer patients using benzodiazepines, possibly due to their influence on kinase signaling or mitochondrial stress responses (O'Donnell et al., 2019). Our findings add mechanistic depth to these observations, proposing PTK2 as a potential mediator linking lorazepam exposure to cancer cell biology. This connection warrants further pharmacological and clinical investigation, particularly regarding the off-target effects of benzodiazepines on tumor growth and treatment response.

From a therapeutic standpoint, PTK2 emerges as a promising biomarker and druggable target in ovarian cancer (Sulzmaier et al., 2014). Several small-molecule inhibitors of PTK2, such as defactinib (VS-6063) and GSK2256098, have demonstrated antitumor efficacy in preclinical and early clinical studies (Mohanty et al., 2020). These agents not only suppress tumor proliferation and metastasis but also modulate the immune microenvironment, enhancing the effectiveness of immune checkpoint inhibitors and chemotherapy (Jiang et al., 2016). Given our findings that PTK2 is associated with immune activation, stromal remodeling, and spatially restricted malignant regions, therapeutic inhibition of PTK2 could potentially disrupt tumor–stroma crosstalk and restore immune accessibility (Serrels et al., 2015). Furthermore, understanding the pharmacological crosstalk between psychiatric medications like lorazepam and oncogenic kinases could provide valuable insights into unintended drug–gene interactions and open avenues for repurposing existing compounds in oncology (Pushpakom et al., 2019).

Several limitations should be acknowledged. First, most of our omics findings are based on associations derived from public databases and computational deconvolution, which cannot fully establish causality between PTK2-related signatures and clinical outcomes; residual confounding factors (e.g., tumor purity and heterogeneity) may also influence correlation-based analyses. Second, immune infiltration estimates vary across algorithms, and although we focused on consistent directional trends, quantitative differences across methods remain unavoidable. Third, the lorazepam–ovarian cancer connection in this study was primarily inferred from target-database integration and network analyses, and direct experimental evidence linking lorazepam exposure to PTK2 regulation (e.g., dose–response, rescue assays, or pathway-level perturbation) is still needed. Fourth, spatial and single-cell results were obtained from available datasets and may not fully capture inter-patient variability or broader clinical subtypes. Finally, although we performed *in vitro* functional validation of PTK2 and supplemented protein-level evidence using UALCAN (CPTAC) and HPA immunohistochemistry, additional mechanistic and multi-layer validation would further strengthen the interpretation. In particular, PTK2 methylation and more comprehensive proteomic validation (ideally together with independent patient cohorts and *in vivo* models) remain important directions for future work.

In summary, this study provides integrative evidence that PTK2 functions as a central oncogenic driver and pharmacological mediator in ovarian cancer. Through multi-omics and experimental validation, we demonstrate that PTK2 not only supports tumor cell proliferation and migration but also orchestrates immune regulation, metabolic reprogramming, and spatial organization within the tumor microenvironment. These findings bridge pharmacological and oncological perspectives, suggesting that targeting PTK2 may serve dual purposes: suppressing tumor progression and enhancing immune responsiveness. Future studies integrating pharmacogenomics, clinical trials, and mechanistic experiments are warranted to further elucidate the therapeutic potential of PTK2 and its interaction with drugs such as lorazepam, ultimately advancing precision oncology and rational drug repositioning strategies in ovarian cancer.

## 5 Conclusion

In this study, we integrated target prediction, multi-omics analyses, and *in vitro* experiments to characterize the role of PTK2 in ovarian cancer. PTK2 emerged as a shared molecular node between lorazepam-related signaling and ovarian cancer and was associated with proliferative, immune, metabolic, and stromal remodeling features, with enrichment in malignant epithelial and fibroblast compartments. These findings support PTK2 as a biomarker and potentially druggable target in ovarian cancer and suggest that PTK2-directed strategies may provide new opportunities for precision therapy and rational drug repurposing.

## Data Availability

The data presented in the study are publicly available from TCGA (The Cancer Genome Atlas) and GEO (Gene Expression Omnibus) repositories. The single-cell RNA-seq dataset OV_EMTAB8107 was obtained from ArrayExpress. The spatial transcriptomics data (GSM6177614) was retrieved from GEO. No novel datasets were generated requiring deposition. All analytical code used in this study is available upon request from the corresponding author.
