# Supplementary figures and images for: Integrative multi-omics and experimental analyses implicate PTK2 as a lorazepam-associated biomarker and potential therapeutic target in ovarian cancer
Source: Front Pharmacol. 2026 Jan 23;16:1744802. doi: 10.3389/fphar.2025.1744802 (PMC12876144; doi:10.3389/fphar.2025.1744802)

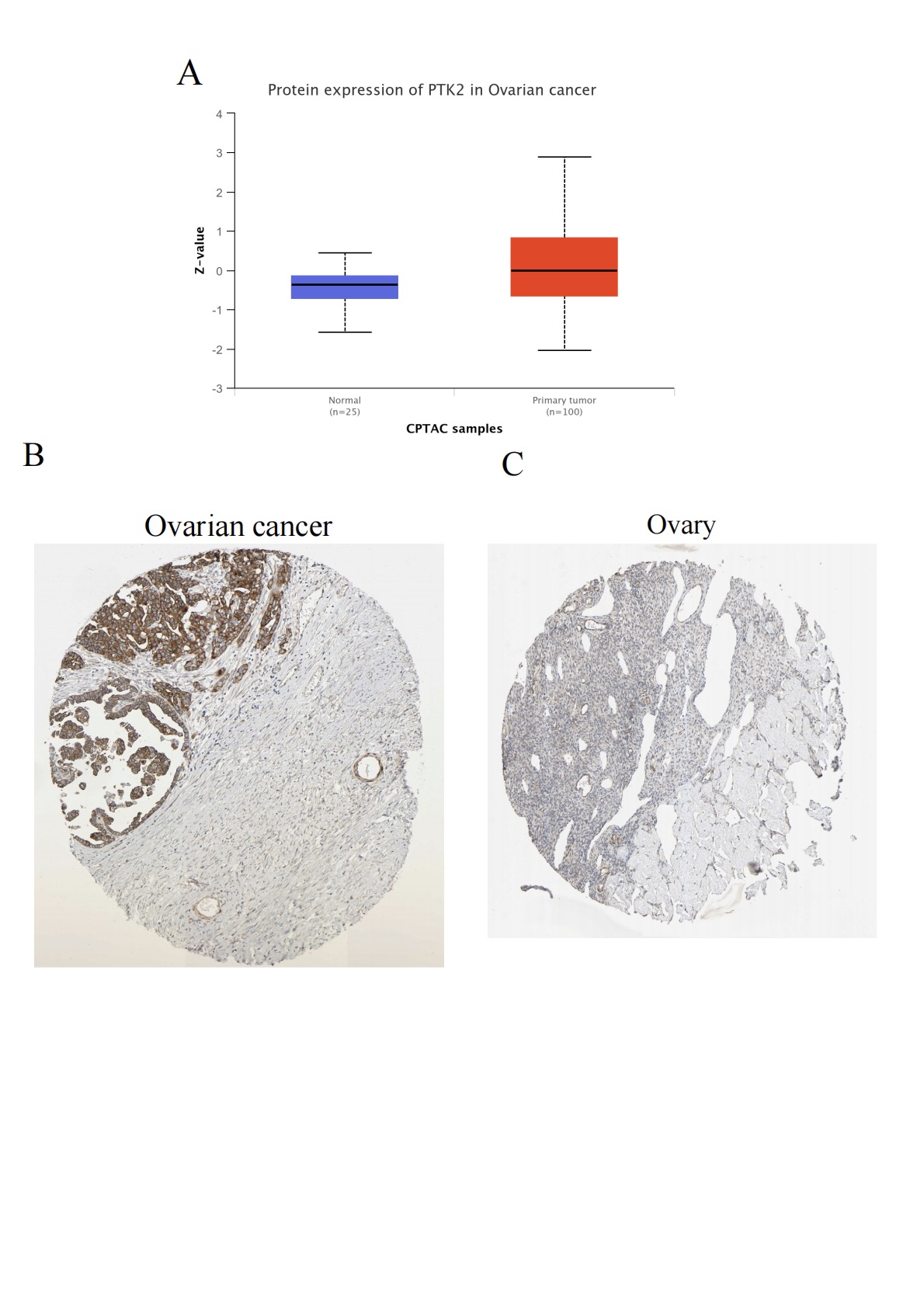

Supplement: Supplementary file 1 [file Image1.jpeg]
